# The evaluation and enhancement strategies of core competencies for older adult caregivers in integrated medical and older adult care institutions

**DOI:** 10.3389/fpubh.2024.1407496

**Published:** 2024-06-18

**Authors:** Chao Rong, Qun-Hong Wu, Hong-Yan Xu, Ming Chang, Lan Zhang, Rong-Rong Xie

**Affiliations:** ^1^School of Humanities and Management, Zhejiang Chinese Medical University, Hangzhou, China; ^2^School of Health Management, Harbin Medical University, Harbin, China; ^3^School of Law, Hangzhou City University, Hangzhou, China

**Keywords:** nursing care, caregivers, eldercare, aging, IADL

## Abstract

The study aimed to understand the main skills of older adult caregivers and find ways to improve these skills. We selected participants using a method called random cluster sampling, where caregivers from 17 different medical and nursing care facilities across seven districts in Hangzhou were chosen. We collected 492 valid questionnaires and conducted interviews with 150 people. To analyze the data, we used *T*-tests and Analysis of Variance (ANOVA) to identify what factors affect caregivers’ skills. We also performed multiple regression analysis to explore these factors in more depth. The analysis showed that age (*p* = 0.041), annual income (*p* < 0.001), and having a training certificate (*p* < 0.001) significantly influence the skills of older adult caregivers. Specifically, caregivers’ age and whether they had a training certificate were linked to how competent they were, with income being a very strong factor. The study highlighted a gap between the caregivers’ current skills and the skills needed for high-quality care. This gap shows the need for training programs that are specifically tailored to the caregivers’ diverse needs and cultural backgrounds. Medical and eldercare facilities should adjust their work and educational programs accordingly. It’s also important to look at how caregivers are paid to make sure their salary reflects their skills and the quality of care they provide. Finally, it’s crucial to integrate a comprehensive training program that leads to certification within eldercare organizations.

## 1 Introduction

By 2022, there will be 771 million people aged 65 and over, accounting for nearly 10% of the global population. This demographic group is expected to increase to 16% by 2050, when there will be about 1.5 billion people aged 65 and over globally (1, 2). According to data from the Seventh National Population Census released by the National Bureau of Statistics, as of November 1, 2020, the older adult population aged 60 and above, and 65 and above in China is substantial, constituting 18.7 and 13.5% of the population, respectively (3). With the burgeoning older adult demographic, there is a concurrent rise in the prevalence of chronic conditions detrimental to their quality of life, such as hypertension, diabetes, and coronary heart disease. This uptick is in stark contrast to the lagging traditional models of eldercare, revealing a profound discrepancy between the escalating needs of the aging populace and the existing caregiving frameworks.

The rapid development of integrated medical and eldercare institutions is a response to this demographic shift. These institutions, as their designation suggests, are equipped with both medical and caregiving capabilities, offering a range of services including psychological comfort, daily life care, palliative care, and medical assistance to the older adult (4). They mitigate to some extent the societal pressures brought about by population aging. Older adult caregivers, individuals who provide services such as daily living and hygiene care for the older adult, serve as one of the primary workforces in these integrated facilities. Their core competencies are crucial to the effective functioning of integrated medical and eldercare institutions in China and significantly influence the quality of eldercare services (5). There is a general lack of older adult care staff in various institutions in China. Older adult care staff are generally older adult and have lower levels of education, and their overall core competencies are relatively low. The most lacking professional medical and mental care skills are exactly what the older adult need the most (6). Therefore, the systemic evaluation and enhancement of caregiver competencies transcend operational considerations and constitute a societal mandate. Nonetheless, there is an evident gap in comprehensive research that specifically addresses the assessment and improvement of these competencies within the Chinese context.

Scholarly investigations into family caregiving dynamics reveal a taxonomy of care necessity, ranging from no need—where the older adult individual is fully independent—to maximum need, necessitating full-time caregiver involvement (7). Research elucidates that the efficacy of respite care—a service that offers temporary relief to primary caregivers—is substantially bolstered by a constellation of factors. These encompass the caregiver’s senior age (65 years or older), marital status (currently married), chronic health conditions, current and deteriorating health status, along with a long-term commitment to caregiving, spanning a decade or more. Furthermore, the health condition of the older adult care recipient and their proficiency in Instrumental Activities of Daily Living (IADL) scores are also pivotal to the success of respite interventions (8). Strategies aimed at fortifying caregivers’ confidence in their care recipients’ ability to function independently may decelerate the trajectory of functional decline in elders receiving home care (9). While extant literature has shed light on the care demands of the older adult and has pinpointed factors that amplify care effectiveness, there remains a conspicuous gap in comprehensive explorations focused on augmenting the foundational competencies of older adult caregivers—a crucial element for advancing the quality of eldercare.

The cultivation of a high-quality, professional workforce of eldercare caregivers has emerged as a focal point of scholarly interest in recent years. Hangzhou, the capital of Zhejiang Province, plays an important role in China’s economic and social development, and has always been at the forefront of integrating modern healthcare and older adult care systems to meet the needs of an aging population. By focusing on Hangzhou, this study aims to utilize the insights of this city to showcase the challenges and progress of older adult care, provide a template, and provide information for broader practices and policies across China. This study will take Hangzhou as a case in point and commence by examining the “core competencies of older adult caregivers,” assessing the current working conditions of caregivers within integrated medical and eldercare institutions in Hangzhou. It aims to summarize the challenges encountered in their roles, analyze the underlying causes, and investigate pertinent influencing factors. Based on the findings, the study will propose viable pathways for enhancing the core competencies of these vital care providers.

## 2 Methods

### 2.1 Research subject

Using a random cluster sampling method, a survey was conducted from July to August 2021, involving a total of 17 medical and older adult care institutions in seven districts of Hangzhou City, including Gongshu District, Binjiang District, Xiaoshan District, Shangcheng District, Qiantang New District, Fuyang District, and Xihu District. The survey targeted the caregivers working in these institutions. This study distributed a total of 500 questionnaires, excluded 8 invalid ones, and collected 492 valid questionnaires, resulting in an effectiveness rate of 98.4%. Using random sampling, a subset of older adult caregivers was selected for semi-structured interviews in addition to the questionnaire survey of older adult caregivers. A total of 150 interviews were conducted. A total of 2 university professors and 6 college students conducted on-site surveys, conducting one-on-one interviews with older adult care workers, collecting questionnaires on site, and reviewing the validity of the questionnaires on the same day. Two college students simultaneously used epidata 3.1 software to perform dual track data entry and proofreading, ensuring the authenticity and reliability of the data.

### 2.2 Research instruments

The survey questionnaire was adapted based on the “Competency Assessment Index System for Nursing Staff in Medical and Older Adult Care Institutions” developed by scholars such as Che et al. (10). Ultimately resulting in the “Core Competency Survey Questionnaire for Nursing Staff in Medical and Older Adult Care Institutions in Hangzhou.” It comprises five sections, including general demographic information, nursing skills, personal qualities, caregiving knowledge, and occupational evaluation.

The general demographic information section includes 10 items, such as the respondent’s hometown, marital status, education level, certification for the position, annual income, duration of training, and years of experience as a caregiver, etc. The caregiving knowledge dimension comprises six items, including knowledge related to the prevention of chronic diseases, safe medication guidance, observation and care of common older adult ailments, knowledge about infectious disease prevention, older adult care-related laws and regulations, and end-of-life care-related knowledge. The nursing skills dimension encompasses 13 items, including skills related to older adult hygiene and sanitation care, older adult nutrition and dietary skills, older adult sleep care, older adult excretion care, pain relief and care for the older adult, hot and cold compress care, nursing skills (use of assistive devices, etc.), basic first aid knowledge and skills (hemostasis, etc.), disinfection and isolation skills, end-of-life care skills, knowledge and skills related to writing nursing records, basic knowledge of traditional Chinese medicine consumption, and methods for preparing Chinese herbal decoctions, as well as the use of healthcare and older adult care apps, totaling 13 items. The personal qualities dimension covers eight items, including an understanding of the psychological characteristics of the older adult, mastery of communication skills with the older adult, respect for and protection of the interests of the older adult, possession of good professional ethics and personal qualities, having good interpersonal relationships and teamwork spirit, having a sound sense of right and wrong, life values, and worldviews, having a rigorous work style, self-discipline, and strong execution capability, and possessing a sound personality. The occupational evaluation section includes three items, which assess aspects such as compensation and benefits, workload, and overall job satisfaction.

Each dimension has three options: complete mastery (2 points), partial mastery (1 point), no mastery (0 points). The care knowledge dimension has a full score of 12 points; the nursing skill dimension has a full score of 26 points; the personal quality dimension has a full score of 16 points. The sum of scores from these three dimensions is the core competency score. A higher score indicates stronger core competency in nursing staff. The Cronbach’s alpha coefficient for the entire questionnaire is 0.916, while the Cronbach’s alpha coefficients for the various dimensions range from 0.788 to 0.867. The overall content validity index (S-CVI) of the questionnaire is 0.902, and the item-level content validity indices (I-CVI) range from 0.779 to 0.871.

The interview outline consists of four questions: (1) How long have you been working as a caregiver? (2) Did you receive any relevant training before officially taking up the position? What was included in the training? (3) How many hours do you work per day? Do you have weekends and holidays off? (4) What are the biggest difficulties and confusions you encounter at work?

### 2.3 Research methodology

The collected data was coded and analyzed using SPSS25.0 software. Descriptive analysis was performed on the demographic data of the survey participants. The scores of the older adult caregivers’ various dimensions and core competencies were subjected to descriptive statistics. *T*-tests and one-way ANOVA were used to explore the factors affecting core competencies. Multiple regression analysis was employed to further analyze the factors influencing the core competencies of older adult caregivers.

## 3 Results

### 3.1 General demographic data of older adult caregivers

Among the participants, there were 98 males (19.92%) and 394 females (80.08%). The majority of the older adult caregivers fell within the age range of 50–60 years, comprising 308 individuals (62.60%). A significant proportion of them came from rural areas, accounting for 448 individuals (91.06%). Marital status indicated that 479 individuals were married (97.36%), while 13 individuals were not married (2.64%). The overall educational attainment of eldercare caregivers is currently at a low level, 286 individuals had attained a primary school education or below (58.13%), and 202 individuals had completed middle school or higher (41.06%). The majority of participants reported an annual income within the 3–5 thousand range, constituting 304 individuals (61.79%). A large portion of the respondents had undergone training, with 454 individuals having received training (92.28%). Among those, 306 individuals had obtained training qualification certificates (62.20%). For the duration of training, 192 individuals received training for less than 7 days (39.02%). Regarding work experience, the highest proportion had more than 36 months of work experience, totaling 266 individuals (54.07%) (Table 1).

**Table 1:** General demographic data of older adult caregivers in Hangzhou integrated healthcare and older adult care facilities (*n* = 492).

Basic information	Group	Number of cases	Proportion (%)
Gender	Male	98	19.92
	Female	394	80.08
Age	Under 50 years old	80	16.26
	Aged 50 to 60 years	308	62.60
	Above 60 years old	104	21.14
Household registration	Rural area	448	91.06
	Urban area	44	8.94
Marital status	In marriage	479	97.36
	Non-in marriage	13	2.64
Educational level	Elementary school and below	286	58.13
	Middle school (junior high, senior High, and vocational school)	202	41.06
	College degree, bachelor’s degree, and higher	4	0.81
Annual income	Less than 30,000	19	3.86
	30,000 to 50,000	304	61.79
	50,000 and above	169	34.35
Training qualification certificate	Yes	306	62.20
	No	186	37.80.
Receiving training	Yes	454	92.28
	No	38	7.72
Number of days of training	Less than 7 days	192	39.02
	8 to 15 days	120	24.39
	More than 16 days	180	36.59
Years of employment	Less than 36 months.	226	45.93
	More than 36 months	266	54.07

### 3.2 Scores on older adult caregiver dimensions and core competencies

The analysis results indicated that the core competencies of older adult caregiver in the surveyed medical and older adult care institutions have an average score of 41.21 ± 8.88, with a maximum score of 54 and a minimum score of 13. Within the three surveyed dimensions: caregiving knowledge, nursing skills, and personal qualities, the dimension with the highest score is personal qualities, with an average score of 15.04 ± 1.63. In this dimension, the maximum score is 16, and the minimum score is 7. For the other two dimensions, caregiving knowledge has an average score of 7.93 ± 3.03, while nursing skills have an average score of 18.24 ± 5.28 (Table 2).

**Table 2:** Scores on various dimensions and core competencies of older adult caregivers.

Dimensions	Number of items	Full score	Mean ± standard deviation	Maximum value	Minimum value
Caregiving knowledge	6	12	7.93 ± 3.03	12	0
Caregiving skills	13	26	18.24 ± 5.28	26	5
Individual attributes	8	16	15.04 ± 1.63	16	7
Core competencies	27	54	41.21 ± 8.88	54	13

### 3.3 Univariate analysis of older adult caregivers core competencies under general demographic data

Independent sample t-tests and one-way analysis of variance (ANOVA) were used to conduct differential analysis on variables including gender, age, educational level, household registration, annual income, marital status, possession of a training certificate, participation in training, training duration, and years of work. According to the results, age, annual income, possession of a training certificate, participation in training, and training duration all exhibited significant differences (*p* < 0.05) (Table 3).

**Table 3:** Univariate analysis of core competencies of older adult caregivers under general demographic data.

Variables	Group	Core competencies (Mean ± SD)	*t*/*F*	*P*
Gender	Male	40.97 ± 8.96	−0.298	0.765
	Female	41.27 ± 8.88		
Age	Under 50 years old	40.35 ± 7.57	7.411	0.001
	Aged 50 to 60 years	40.43 ± 8.66		
	Above 60 years old	44.14 ± 9.78		
Household registration	Rural area	41.38 ± 9.04	1.673	0.100
	Urban area	39.51 ± 6.80		
Marital status	In marriage	41.21 ± 8.93	−0.049	0.961
	Non-in marriage	41.33 ± 7.02		
Educational level	Elementary school and below	40.65 ± 9.16	1.896	0.151
	Middle school (junior high, senior high, and vocational school)	42.08 ± 8.51		
	College degree, bachelor’s degree, and higher	37.50 ± 3.70		
Annual income	Less than 30,000	47.95 ± 7.54	7.237	0.001
	30,000 to 50,000	41.44 ± 9.06		
	50,000 and above	40.03 ± 8.30		
Training qualification certificate	Yes	44.23 ± 8.56	11.251	<0.001
	No	36.25 ± 7.00		
Receiving training	Yes	41.56 ± 8.96	3.775	<0.001
	No	37.08 ± 6.84		
Number of days of training	Less than 7 days	40.08 ± 7.93	3.378	0.035
	8 to 15 days	42.73 ± 8.28		
	More than 16 days	41.41 ± 9.98		
Years of employment	Less than 36 months.	42.05 ± 9.61	3.790	0.052
	More than 36 months	40.49 ± 8.13		

### 3.4 There is a positive correlation between individual quality and job satisfaction of older adult caregivers

One-way analysis of variance (ANOVA) was employed to conduct differential analysis on salary and compensation assessment, workload evaluation, and job satisfaction assessment. The statistical results revealed that there were no significant differences in salary and compensation assessment and workload evaluation (*p* > 0.05). However, there was a significant difference in job satisfaction assessment (*p* < 0.05) (Table 4).

**Table 4:** Univariate analysis of occupational appraisal and older adult caregivers individual quality dimensions.

Variables	Group	Individual quality dimensions (Mean ± SD)	*t*/*F*	*P*
Salary and compensation assessment	High	13.50 ± 0.92	0.921	0.399
	Moderate	13.21 ± 1.39		
	Low	13.06 ± 1.63		
Workload evaluation	Heavy	13.36 ± 1.10	1.455	0.234
	Moderate	13.11 ± 1.54		
	Light	13.25 ± 1.65		
Job satisfaction assessment	Satisfied	13.38 ± 1.18	7.415	0.001
	Neutral	12.96 ± 1.67		
	Dissatisfied	12.20 ± 2.15		

### 3.5 Multivariate regression analysis of factors influencing older adult caregivers core competencies

Multiple regression analysis was conducted using the total score of older adult caregiver core competencies as the dependent variable, and the factors that showed statistical significance in the independent sample t-tests and one-way analysis of variance as independent variables: age, annual income, possession of a training certificate, participation in training, and training duration, as outlined in Supplementary Table S1. The statistical results indicate that age, annual income, and possession of a training certificate are the primary influencing factors on older adult caregiver core competencies (Table 5).

**Table 5:** Results of multivariate regression analysis on factors influencing older adult caregivers core competencies.

Independent variable	*β*	95%CI	*t*	*P*
Age	1.210	0.039 to 2.379	2.047	0.041
Annual income	2.632	−3.965 to −1.318	3.904	<0.001
Obtaining a training certificate	8.042	−9.493 to −6.522	10.658	<0.001
Receiving training	0.158	−2.924 to 2.551	0.113	0.910
Number of training days	0.463	−0.355 to 1.275	1.117	0.264

### 3.6 Interview results of older adult caregiver

In response to the question “What is your biggest challenge in your current job?” the most common answers include: low educational level and inability to write; limited rest time; insufficient sleep; lack of understanding from older adult individuals and their families; lack of support from management; occasionally neglecting personal health due to caring for older adult individuals with limited mobility; and difficulties in dealing with older adult individuals with irritable temperaments.

## 4 Discussion

The analysis reveals that caregivers in Hangzhou’s eldercare sector possess an average care knowledge proficiency score of 7.93 out of a potential 12. This suboptimal performance is influenced significantly by the caregivers’ educational backgrounds, primarily those from rural areas with limited formal education. Such a deficit is particularly problematic in managing chronic diseases, a critical area in geriatric care. These findings suggest a pressing need for educational programs tailored to bridge knowledge gaps, particularly in chronic disease management, to enhance caregiver competencies. Another layer of complexity is added by the caregivers’ origins—predominantly from provinces like Anhui and Henan—where distinct linguistic and cultural norms may interfere with the effectiveness of current training modalities. The presence of such barriers indicates that training programs must not only be informative but also culturally and linguistically adapted to the caregivers’ backgrounds to improve learning outcomes and care quality. The variance in nursing skills, with scores ranging from 5 to 26 out of a possible 26, underscores a significant inconsistency in the mastery of essential nursing techniques. While routine daily care tasks are generally well-managed, there is a notable deficiency in more complex medical procedures, such as the application of hot and cold compresses or the execution of emergency first aid. This gap highlights the necessity for more rigorous and specialized training that aligns with the medical needs of the older adult, ensuring better preparedness among caregivers to handle diverse healthcare scenarios (11). The perception of older adult caregiving within traditional Chinese culture—as a low-skill and low-status occupation—further complicates talent recruitment and retention in this sector (12). This cultural view diminishes the profession’s appeal to potentially skilled workers and perpetuates a cycle of underqualification (13). Addressing these occupational stereotypes and promoting caregiving as a respected and essential profession are vital steps toward attracting and retaining higher-quality talent, which is crucial for improving the overall standard of older adult care.

Our study highlights significant differences in core competencies among eldercare caregivers across various age groups, revealing that those above 60 years of age exhibit the highest competencies. This observation aligns with prior research indicating that older caregivers often report higher self-efficacy (*β* = 0.215, 95%CI 0.139–1.027) (14). This raises important questions about the factors contributing to this age-related disparity in skills and knowledge. Caregivers within the 50–60 age range often face a dual burden of responsibilities. They not only provide care in integrated medical-eldercare settings but also tend to familial obligations, including the care of their own older adult parents and grandchildren. This multiplicity of roles may dilute their ability to focus entirely on their professional responsibilities, potentially leading to lower competency scores. This phenomenon suggests that life-stage pressures can significantly impact professional capacity. Conversely, caregivers over the age of 60, often retirees, are likely to have fewer family obligations and a closer affinity with the elder demographic. Their life experiences and proximity in age may foster a deeper empathy and commitment to their caregiving roles, enhancing their effectiveness and competencies in eldercare. This demographic’s unique position presents an opportunity for optimizing care practices within professional settings. Given the impact of age on caregiver competencies, it is crucial for integrated medical-eldercare institutions to tailor work assignments and training modules to suit the diverse needs and capabilities of caregivers at different life stages. By doing so, institutions can enhance the overall quality of care and ensure that caregivers are supported in balancing personal commitments with professional development (15). Future training programs and organizational policies must consider these demographic differences to optimize caregiver efficacy and sustain high standards of care across all age groups.

Our study investigates the significant intersection between income remuneration and core competencies among eldercare caregivers. Empirical data demonstrate a direct correlation between caregivers’ annual income and their skills essential for high-quality geriatric care. Caregivers earning higher incomes tend to display a broader array of core competencies, which aligns with previous research indicating that income rewards are linked to positive attitudes and professional growth in caregiving roles (*β* = 0.214, 95%CI: 0.117–1.461) (14).The findings suggest that income satisfaction plays a crucial role in fostering caregivers’ dedication and enthusiasm for their roles. Higher earnings are correlated with a greater willingness among caregivers to engage in ongoing professional development and skill enhancement. This inclination toward lifelong learning is critical in the dynamic field of eldercare, where evolving standards require continuous educational engagement. The relationship between income satisfaction and the pursuit of professional excellence underscores the need for equitable and motivating compensation strategies. These strategies should not only ensure a decent living wage reflecting the complexity of caregiving tasks but also aim to improve the overall standard of care provided to the older adult. It is imperative for eldercare institutions and policymakers to invest in their workforce through fair compensation practices. In light of these insights, it is critical for eldercare service providers to reassess their current pay structures. Implementing salary scales that reflect the caregiver’s level of expertise and the quality of care they provide could initiate a virtuous cycle (16)^.^ Enhanced caregiver competencies, driven by better income incentives, could lead to higher-quality eldercare services, justifying further income and attracting a more skilled and motivated workforce to the sector.

Our research demonstrates the profound impact of structured training programs and subsequent certification on the competencies of eldercare caregivers. Those who have undergone formal training and obtained certification exhibit markedly higher competency levels compared to their untrained peers. This correlation between certification and enhanced professional acumen underscores the value of formal education processes in the eldercare sector. The diversity of both caregivers and the older adult population in integrated medical-eldercare institutions poses unique challenges. Caregivers come from various backgrounds, ranging from domestic roles to professional careers, which can lead to potential misunderstandings and interpersonal conflicts (17, 18). Targeted training that encompasses both technical skills and soft skills such as effective communication and conflict resolution is crucial to address these challenges. Caregivers with prolonged experience in the field tend to develop and refine their skills continuously, enhancing their ability to manage emergencies and complex caregiving situations. This experiential enhancement of practical skills is essential for improving the overall quality of care. The advantages of certification extend beyond technical caregiving competencies. Certified caregivers are better equipped to navigate social interactions and foster harmonious relationships within the caregiving environment, contributing to a more peaceful and supportive setting for the older adult. The synthesis of vocational skills and interpersonal adeptness is crucial for the superior performance of certified caregivers in eldercare. Given the clear benefits of structured training and certification, eldercare institutions should consider integrating robust training programs with a clear certification pathway into their operational models. Such strategic investments not only enhance caregiver competencies but also improve the overall quality of eldercare. Additionally, the potential integration of artificial intelligence in training programs could further enhance these outcomes by improving the efficiency and adaptability of caregiving practices (19, 20). Investing in caregiver education and certification is not just an institutional requirement but a strategic approach to elevating the standards of eldercare. By embracing comprehensive training models and supporting continuous professional development, eldercare institutions can better meet the complex needs of the aging population and ensure high-quality care.

Our research explores the significant correlation between job satisfaction among eldercare caregivers and enhancements in their personal quality dimensions. Caregivers with higher job satisfaction demonstrate superior understanding of the older adult’s psychological characteristics and adhere more closely to professional ethics. This positive relationship underscores the importance of job satisfaction in fostering personal growth and ethical professional conduct. Job satisfaction in eldercare is influenced by a variety of factors including remuneration, work assignments, interpersonal relations among staff, and the work environment. These factors collectively impact caregivers’ motivation and their ability to provide high-quality care. It is well-established that job satisfaction is a critical buffer against job burnout, which can manifest as reduced enthusiasm, decreased interaction with the older adult, and a shift toward self-interest at the expense of care recipients’ wellbeing (21). Understanding the link between job satisfaction and burnout provides a pathway for enhancing caregivers’ personal qualities. Institutions play a pivotal role in this process by proactively addressing factors that contribute to job satisfaction. Measures that improve job satisfaction can lead to reduced burnout and enhanced personal and professional qualities among caregivers, ultimately improving the quality of care provided to the older adult. Recent advancements in technology, especially the integration of artificial intelligence in eldercare, offer significant potential to support caregivers. By reducing the workload and automating routine tasks, AI can help caregivers focus more on personalized care aspects, enhancing job satisfaction and retention (22). This technological support is not just a tool for efficiency but also a strategic asset in improving the caregiving environment and staff wellbeing. The evidence suggests that eldercare institutions must engage in active reforms aimed at bolstering caregiver satisfaction. Such reforms might include policy changes, improved communication channels, and a supportive work environment that fosters a sense of value and belonging among caregivers. By investing in caregiver wellbeing, institutions indirectly enhance the quality of care provided to the older adult, with substantial implications for the future of eldercare services.

## 5 Conclusion

The disparity between the skills that are necessary for effective eldercare and those currently exhibited by caregivers not only impacts the quality of care but also highlights the critical need for specialized and culturally attuned training programs. The effectiveness of caregiver training programs greatly depends on their relevance to the specific demands of the caregiving role and the cultural context in which care is provided. Moreover, given the significant influence of a caregiver’s age on their core competencies, it is imperative for integrated medical-eldercare institutions to develop role-specific training that considers the life stage and career phase of each caregiver. Compensation is another critical factor influencing caregiver performance and retention. Eldercare providers need to reassess their existing pay structures to ensure they are aligned with the caregivers’ skill levels and the quality of care they are capable of delivering. The strategic importance of well-defined training and certification paths in eldercare cannot be overstated. Institutions must adopt robust training programs that culminate in certification, thereby assuring a standard level of competency across all caregivers. In addition, the incorporation of technology and modern educational methodologies, such as the use of artificial intelligence in training programs, can further enhance these outcomes by improving the efficiency and adaptability of caregiving practices.

### 5.1 Limitations and implications

This study has the following limitations. Firstly, random cluster sampling may increase sampling error. Individuals within each cluster often exhibit high similarity, while there may be significant differences between clusters. As a result, this method may not represent the overall population as effectively as simple random sampling. Secondly, in this study, a cross-sectional survey was conducted to investigate the core competencies of older adult caregivers in integrated medical and older adult care institutions. The conclusions drawn from this approach are limited in their ability to infer causality. This study has the following implications. Firstly, customized training programs. Institutions should develop and implement training programs that are customized to the life stages and cultural backgrounds of caregivers. This approach not only enhances caregiver competencies but also improves job satisfaction and retention. Secondly, revision of compensation structures. Based on the findings, eldercare services should revise their compensation structures to better align with the skills and the quality of care provided by caregivers. This would motivate caregivers to pursue further professional development and improve the quality of care. Lastly, technological advancements in caregiving. Practical application of AI and other technologies can reduce the physical and cognitive load on caregivers, allowing them to focus more on the interpersonal aspects of care. Institutions should consider investing in such technologies to enhance the efficiency and effectiveness of caregiving.

## Data availability statement

The raw data supporting the conclusions of this article will be made available by the authors, without undue reservation.

## Ethics statement

The studies involving humans were approved by the Medical Ethics Committee of Zhejiang Chinese Medical University. The studies were conducted in accordance with the local legislation and institutional requirements. The participants provided their written informed consent to participate in this study.

## Author contributions

CR: Conceptualization, Data curation, Writing – original draft. Q-HW: Investigation, Writing – review & editing. H-YX: Writing – review & editing, Validation. MC: Conceptualization, Formal analysis, Writing – original draft. LZ: Formal analysis, Writing – review & editing. R-RX: Conceptualization, Writing – review & editing.
