# Supplementary material for: The evaluation and enhancement strategies of core competencies for older adult caregivers in integrated medical and older adult care institutions
Source: Front Public Health. 2024 Jun 18;12:1407496. doi: 10.3389/fpubh.2024.1407496 (PMC11217317; doi:10.3389/fpubh.2024.1407496)
Supplement: Supplementary file 1 [file Table_1.docx]

Supplementary Table 1 Assignments of each variable

| Independent variable | Variable assignment |
| --- | --- |
| Age | Below 50 years old = 1, 50-60 years old = 2, above 60 years old = 3 |
| Annual income | Below 30,000 RMB = 1, 30,000-50,000 RMB = 2, above 50,000 RMB = 3 |
| Obtaining a training certificate | Yes = 1, No = 2 |
| Receiving training | Yes = 1, No = 2 |
| Number of training days | Less than 7 days = 1, 8-15 days = 2, More than 16 days = 3 |
